# Cholécystite aigue gangréneuse alithiasique en postopératoire d’une chirurgie orthopédique: à propos d’un cas

**DOI:** 10.11604/pamj.2017.27.8.11526

**Published:** 2017-05-02

**Authors:** Hichem Cheikhrouhou, Karim Jmal, Amine Kharrat, Meriem Keskes, Abdelhamid Karoui

**Affiliations:** 1 Département d’Anesthésie, CHU Habib Bourguiba, Sfax, Tunisie

**Keywords:** Cholécystite alithiasique gangreneuse postopératoire, diagnostic, cholécystectomie, chirurgie orthopédique, Postoperative acalculous gangrenous cholecystitis, diagnosis, cholecystectomy, orthopaedic surgery

## Abstract

La cholécystite gangreneuse alithiasique postopératoire est une complication grave et sévère, surtout chez les malades hospitalisés en réanimation. Elle survient le plus souvent au décours d'une chirurgie vasculaire ou digestive majeure, d'un polytraumatisme, dans un contexte septique ou dans un contexte de choc. Nous rapportons l'observation d'un homme âgé de 74 ans opéré d'une fracture du col du fémur, au sixième jour postopératoire il a développé un tableau clinique d'une cholécystite aigue dont les explorations radiologiques ont confirmé son caractère alithiasique. Après une cholécystectomie en urgence, l'étude anatomopathologique a conclu à une cholécystite gangreneuse alithiasique.

## Abstract (en)

Postoperative acalculous gangrenous cholecystitis is a serious and severe complication, especially in patients hospitalized in the Department of Reanimation. It occurs most often during vascular surgery or major digestive surgery, a polytrauma, in a context septic or in a context of shock. We report the case of a 74 year man who underwent surgery for femoral neck fracture. On the sixth postoperative day, he developed acute cholecystitis. Radiological examinations confirmed acalculous cholecystitis. After emergency cholecystectomy, anatomo-pathologic study confirmed the diagnosis of acalculous gangrenous cholecystitis.

## Introduction

La cholécystite aigue postopératoire alithiasique et gangreneuse a été décrite pour la première fois en 1947 par Glenn, sa fréquence reste difficile à estimer. La définition de cholécystite gangreneuse est anatomopathologique, elle se définit par l'existence d'une nécrose de coagulation qui affecte la muqueuse et la musculeuse. Sa physio-pathogénie est multi factorielle et quand elle est diagnostiquée dans un contexte postopératoire, elle est le plus souvent alithiasique [1].

## Patient et observation

**Figure 1:**
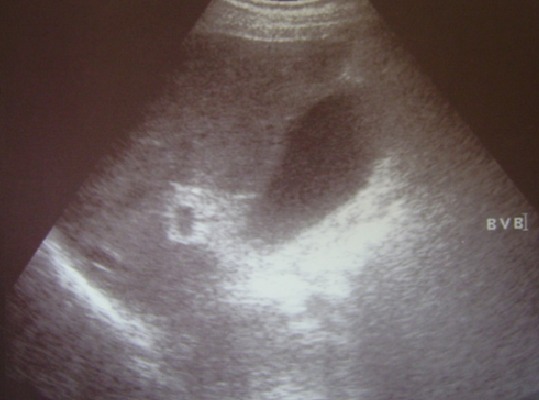
Échographie abdominale: vésicule biliaire alithiasique distendue et à paroi épaissie

**Figure 2:**
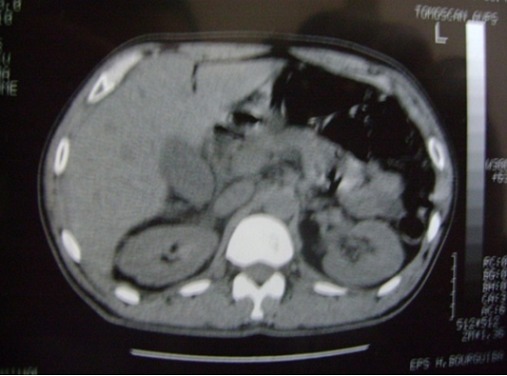
Tomodensitométrie hépato-biliaire: cholécystite alithiasique avec épaississement de la paroi vésiculaire

**Figure 3:**
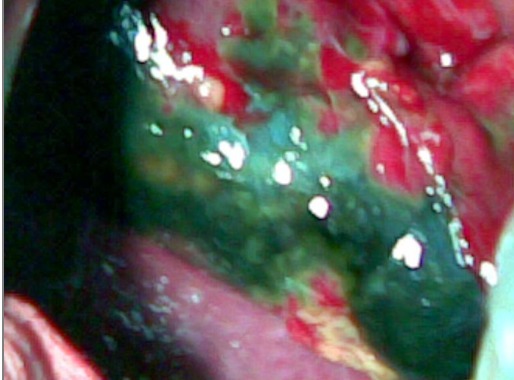
Pièce opératoire de la vésicule biliaire gangrenée: aspect d'une muqueuse ulcérée et hémorragique

Patient âgé de 74 ans avec comme antécédent une hypertension artérielle équilibrée, était victime d'un accident domestique responsable d'une fracture du col du fémur gauche. Le patient a bénéficié d'une chirurgie orthopédique dans les deux premiers jours après l'accident avec suites opératoires simples autorisant le retour à domicile. Au sixième jour postopératoire, le patient a été hospitalisé au service de chirurgie générale pour douleur de l'hypochondre droit sans irradiation et accompagnée par des vomissements. L'examen clinique trouve un patient fébrile à 38.8°c, une pâleur conjonctivale, une tachypnée à 22 cycles/mn et un état hémodynamique stable, à la palpation une sensibilité au niveau de l'hypochondre droit avec défense et un signe de Murphy positif. La biologie a montré une hyperleucocytose à 10300 élément/mm^3^, une hémoglobine à 12,2g/dl, une azotémie à 11,5 mmol/l, une glycémie à 4 mmol/l, l'ASAT à 35UI/ml, l'ALAT à 25UI/ml, la bilirubine totale à 13 μmol/l, une calcémie à 2,1mmol/l, une amylasémie à 41 UI/ml, un TP à 77%, et une CRP positive. L'échographie abdominale a montré une vésicule biliaire distendue, à paroi épaissie mesurant de 6,5mm et de contenu transonore alithiasique (Figure 1), les voies biliaires sont normales, le foie est de taille normale et d'échostructure homogène et absence d'épanchement péritonéal. La tomodensitométrie hépato-biliaire n'a pas objectivé une densité évocatrice de lithiase vésiculaire mais un épaississement de la paroi vésiculaire (Figure 2). Devant ce tableau de cholécystite aigue alithiasique, on a débuté une antibiothérapie à base d'amoxcicilline / acide clavulanique et une cholécystectomie par une incision sous costal droite a été réalisée en urgence. L'exploration peropératoire a montré : Une cholécystite gangreneuse avec abcès péri vésiculaire et une réaction péritonéale. Les gestes effectués : cholécystectomie après ligature section de l'artère cystique et du canal cystique, une cholangiographie peropératoire a montré une voie biliaire principale fine, un bon passage duodénal et absence d'image d'obstacle, un drainage par un drain trans-cystique a été mis en place. L'évolution postopératoire était favorable avec sortie à domicile à j10 post-op et il a été revu deux mois après l'intervention avec bon état clinique. L'examen anatomopathologique de la pièce opératoire trouve sur le plan macroscopique une vésicule biliaire de 9 cm de longueur et de 6 cm de circonférence maximale, sa muqueuse est ulcérée et hémorragique, sa paroi est moyennement scléreuse, sur le plan histologique : une cholécystite en poussée aigue gangreneuse (Figure 3).

## Discussion

Dans la littérature, la cholécystite gangreneuse alithiasique postopératoire survient préférentiellement chez l'homme entre 50 et 70 ans et en particulier sur un terrain vasculaire ou de diabète [2]. Sa fréquence reste difficile à estimer. La prévalence la plus élevée est observée dans les suites des interventions chirurgicales lourdes. Toute intervention chirurgicale peut cependant se compliquer de cholécystite aigue alithiasique (CAA), en particulier la chirurgie digestive qui représente la situation où survient la moitié de l'ensemble des CAA. Dans la série de Richard et al [3], 12 parmi 20 patients (60%) ont développé une CAA en postopératoire, dont 50% ont eu une intervention abdominale en dehors des voies biliaires, et 10% ont eu une intervention orthopédique. Le délai de survenue la cholécystite gangreneuse alithiasique en postopératoire varie de quelques jours à deux mois. Dans une étude récente de Choo SK portant sur 1211 patients âgés de plus de 65 ans et opérés d'une fracture du fémur, il a trouvé neuf cas de Cholécystite aigue postopératoire et il a conclu que ces malades âgés et tarés sont plus exposés à développer cette complication postopératoire [4]. La pathogénie de cette affection est très discutée. Plusieurs théories sont proposées : mécanique (liée à des phénomènes de stase biliaire), infectieux (avec atteinte muqueuse et pariétale), ou vasculaire (en relation avec une ischémie locale). La théorie mécanique serait surtout caractérisée par des troubles de la vidange vésiculaire, la distension du cholécyste peut en effet être induite par l´administration d´un certain nombre d´agents pharmacologiques et, parmi ceux-ci, les antispasmodiques, les antalgiques et certains calmants ou morphiniques rendus souvent nécessaires pour permettre une ventilation assistée satisfaisante [5]. Les opiacés, fréquemment utilisés en réanimation, en induisant un spasme du sphincter d´Oddi favoriseraient également l´hyperpression biliaire. La ventilation mécanique avec une pression expiratoire positive augmente la pression biliaire et diminue le flux biliaire de manière significative. La théorie vasculaire a été évoquée en raison de la fréquence des lésions gangreneuses de la vésicule. L´existence d´une athéromatose diffuse ayant souvent été notée chez ces malades âgés. Certains ont pu penser que les CAA n´étaient en fait qu´une des manifestations de la maladie artérielle. En réalité, les dépôts athéromateux sont exceptionnellement retrouvés dans l'artère cystique. Une origine ischémique est par contre probable dans un certain nombre de cas où furent retrouvés soit une embolie artérielle, une CIVD, une thrombose de l´artère cystique ou plus généralement un phénomène de « bas débit » splanchnique que l´on observe dans les collapsus [6]. Pour la théorie infectieuse Il n´est pas exclu que l´infection puisse agir de façon péjorative sur des parois vésiculaires fragilisées, mais il faut également reconnaître que toute ischémie pariétale est susceptible de générer une pullulation microbienne [7]. Un état septique est de plus fréquemment retrouvé dans les CAA survenant dans les services de réanimation [5]. Les endotoxines bactériennes sont aussi impliquées comme un facteur contributif mais beaucoup de culture de bile des patients avec CAA étaient négatives. Les endotoxines bactériennes induisent chez l´animal une CAA probablement par une activation de différents facteurs de la coagulation.

La cholécystite gangreneuse alithiasique postopératoire pose un difficile problème diagnostique [1], elle se manifeste par une symptomatologie d'emprunt, les signes locaux sont peu marqués, contrastant avec une atteinte de l'état général. Dans la littérature, le taux de positivité de ce signe est de 33% [8]. Dans notre cas, le patient était fébrile avec une douleur de l'hypochondre droit et le signe de Murphy a été positif à l'examen clinique. A l'exception de l'hyperleucocytose, le tableau biologique de cholécystite n'a rien de spécifique. Dans ce cadre les examens radiologiques prennent toute leur importance. Ces examens sont dominés par l'échographie, c'est un examen de première intention en pathologie hépato biliaire, il est anodin, reproductible, très sensible, portable au lit et capable d'identifier d'autres pathologies adjacentes. Elle permet outre le diagnostic d'une cholécystite alithiasique, de suggérer sa nature gangreneuse. En effet, certains signes échographiques sont considérés comme spécifiques de la cholécystite gangreneuse, tels que l'épaississement irrégulier de la paroi vésiculaire, l'aspect stratifié de la paroi, la présence d'une membrane endoluminale et la constatation d'un épanchement péri vésiculaire [9]. Pour notre malade, l'échographie abdominale a montré une vésicule biliaire distendue, à paroi épaissie mesurant de 6,5mm et de contenu transonore et alithiasique. Le scanner n'est pas indispensable pour le diagnostic. Il est généralement pratiqué pour d'autres raisons. Toutefois, il peut signaler des signes spécifiques qui ne peuvent pas être déterminés par l'échographie, telles qu'ne hyperdensité de la paroi ne nécessitant pas l'injection d'un produit de contraste et la présence des gaz dans la paroi ou en intraluminal. C'est un examen radiologique de grande spécificité (96%) pour l'identification de la forme gangreneuse de CA, mais sa sensibilité ne dépasse pas 29% [10]. La scintigraphie décrite dans le cadre de cholécystite aiguë alithiasique par certains auteurs [11]. Cette exploration montre, en cas de gangrène, la présence d'une aire dans laquelle il y a accumulation du radio traceur en péri vésiculaire et extravasation du radio traceur en cas de perforation. Elle présente comme inconvénients sa réalisation difficile. L'imagerie par résonance magnétique est rarement réalisée dans le cadre des cholécystites gangreneuses alithiasiques, elle peut déceler les lésions de la paroi vésiculaire et particulièrement de la muqueuse [12]. L'évolution spontanée de cette pathologie se fait vers les complications graves telles que la perforation et la péritonite biliaire. Une fois le diagnostic de cholécystite gangreneuse alithiasique est posé, la cholécystectomie doit être indiquée en urgence [13].

Cette attitude s'explique par la fréquence de survenue d'une perforation vésiculaire dans l'évolution de cette forme. Selon Johnson, le taux de perforation passe de 8 à 40% si la cholécystectomie est réalisée avant ou après les 48 heures suivant le début des symptômes [14]. Le traitement chirurgical doit être précédé par une préparation médicale rapide basée sur une rééquilibration hydro électrolytique et la correction des tares préexistantes, une antibiothérapie à large spectre couvrant les germes anaérobies et éventuellement une correction des troubles de la crase sanguine. Malgré le progrès et la performance de la voie laparoscopique [15], la conversion par voie chirurgicale est fréquente, vu la fragilité de la paroi vésiculaire et le risque de traumatisme du pédicule hépatique [16], ainsi la voie d'abord sous costale classique reste toujours recommandée pour cette pathologie. L'intervention découvre une vésicule en général tendue, violacée avec des plaques de gangrène n'intéressant pas le plus souvent toute l'aire vésiculaire. La cholécystectomie antérograde retrouve ici sa meilleure indication. Elle permet d'éviter la blessure de la voie biliaire et facilite l'isolement en dernier du canal cystique afin de réaliser une cholangiographie peropératoire. Cette opacification doit éliminer obligatoirement une lithiase de la voie biliaire, passée inaperçue à l'exploration préopératoire, particulièrement chez les sujets présentant un iléus intestinal. La cholécystostomie percutanée s´est imposée comme une thérapeutique de choix chez les malades inopérables. Cette technique permet d´évacuer la bile et de laisser en place un drain vésiculaire pendant un mois, dans la plupart des séries, le geste est pratiquement toujours réalisable avec une morbidité initiale faible [17]. La cholécystite gangreneuse alithiasique postopératoire reste une affection grave avec un taux de mortalité, quatre fois plus élevée que celui de la cholécystite banale. L'âge avancé, les tares cardiovasculaires et le diabète, de même que le retard de diagnostic et de prise en charge chirurgicale, sont les facteurs de risques démontrés d'une mortalité élevée pour cette pathologie [1].

## Conclusion

La cholécystite gangreneuse alithiasique postopératoire est une complication grave et sévère. Elle survient préférentiellement chez les sujets âgés et tarés suite à une intervention chirurgicale. Le diagnostic préopératoire reste dans la majorité des cas incertain malgré les progrès de l'imagerie, donc il faut préconiser d'opérer en urgence toute cholécystite alithiasique diagnostiquée.

## Conflits d’intérêts

Les auteurs ne déclarent aucun conflit d'intérêts.
